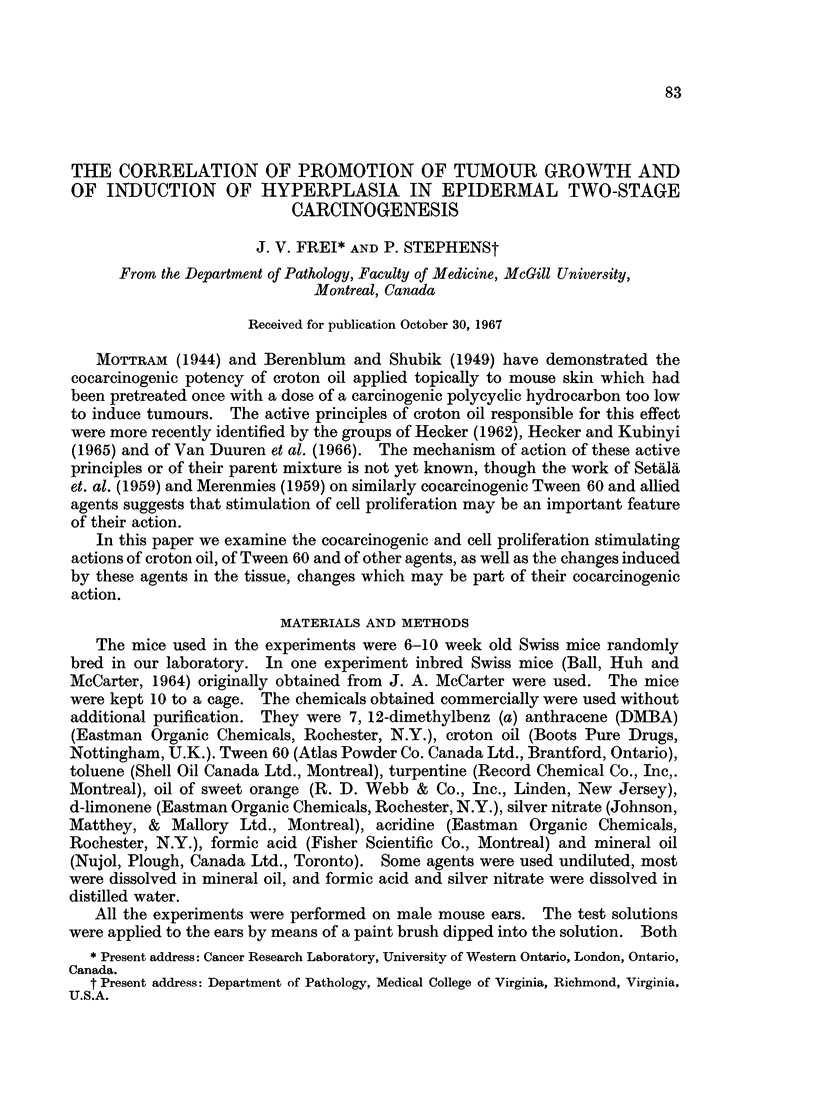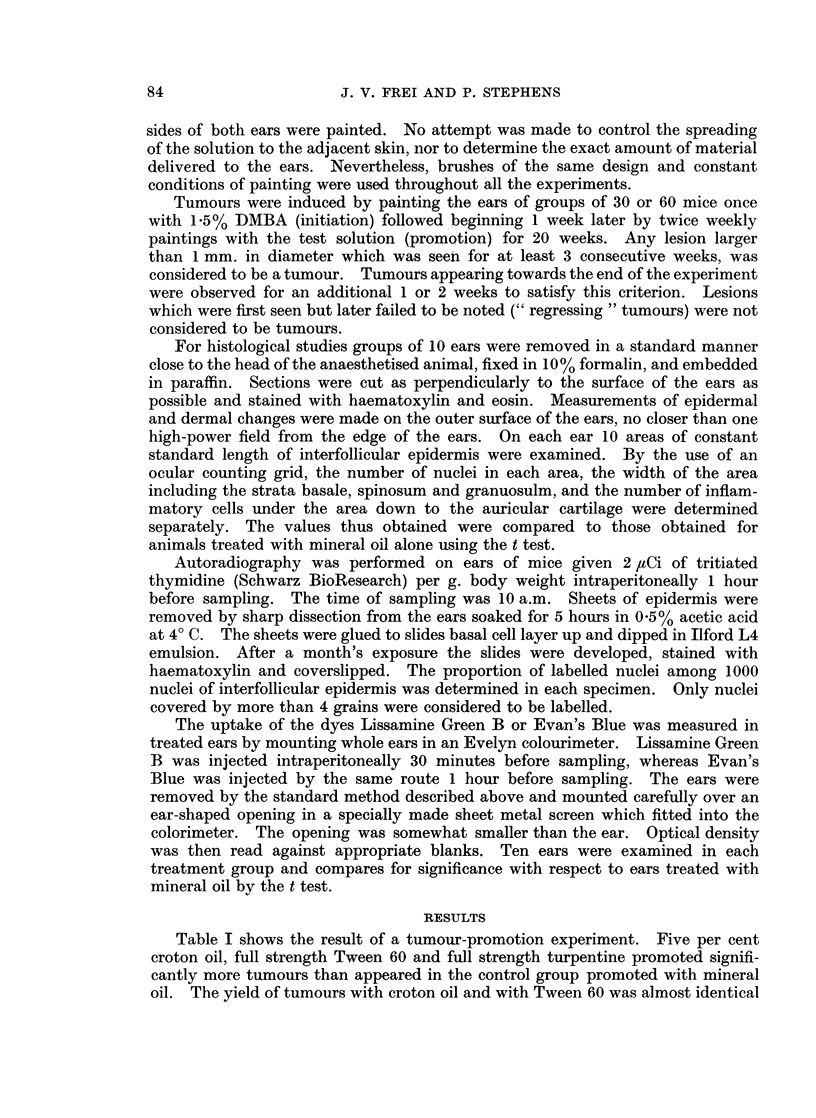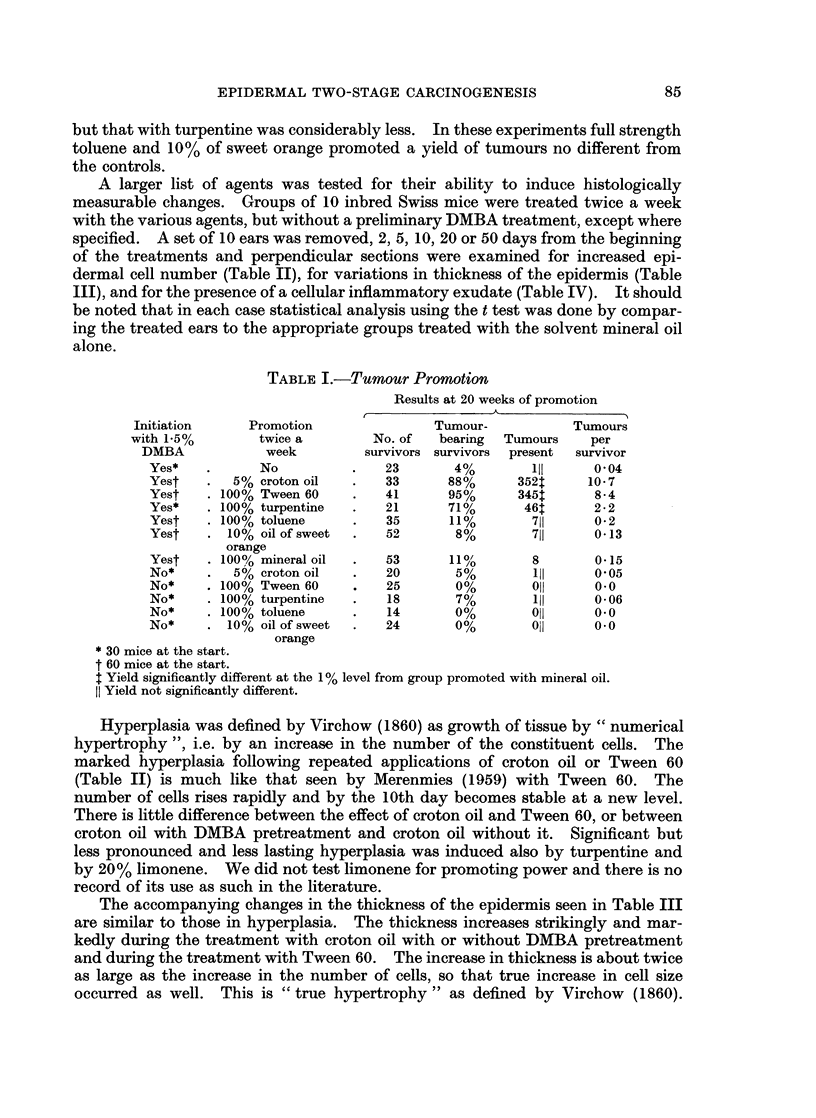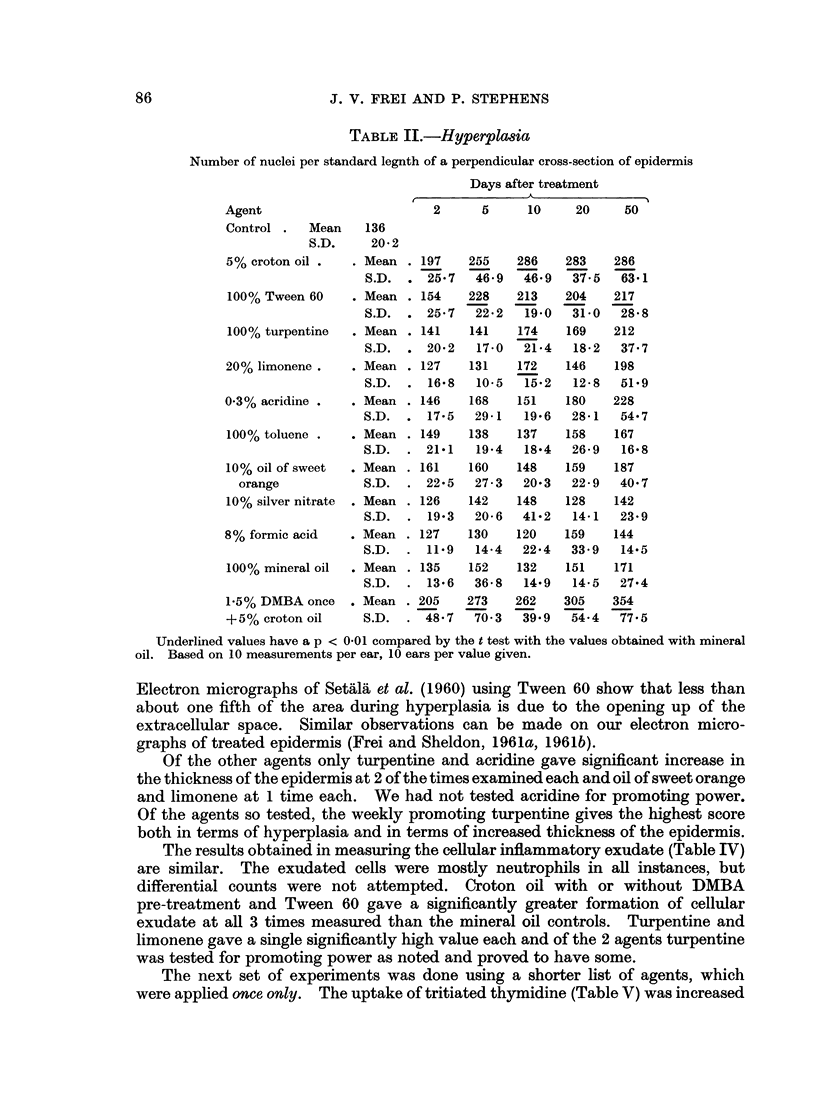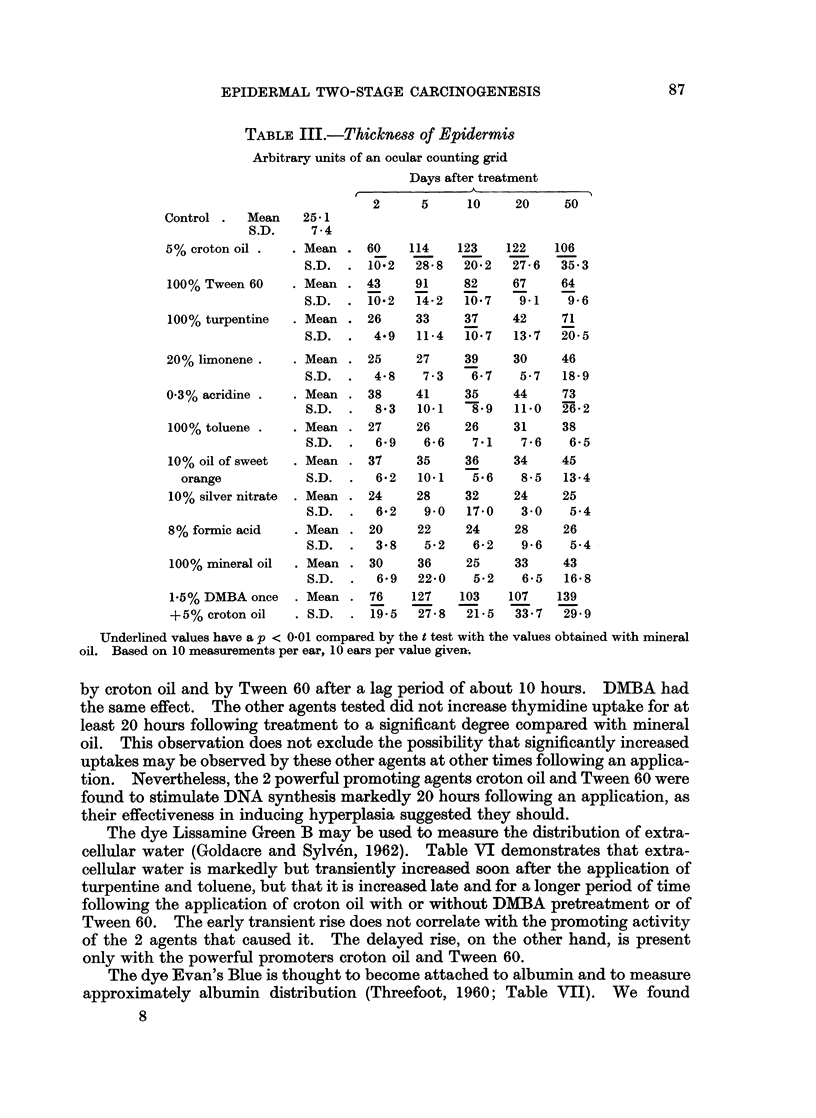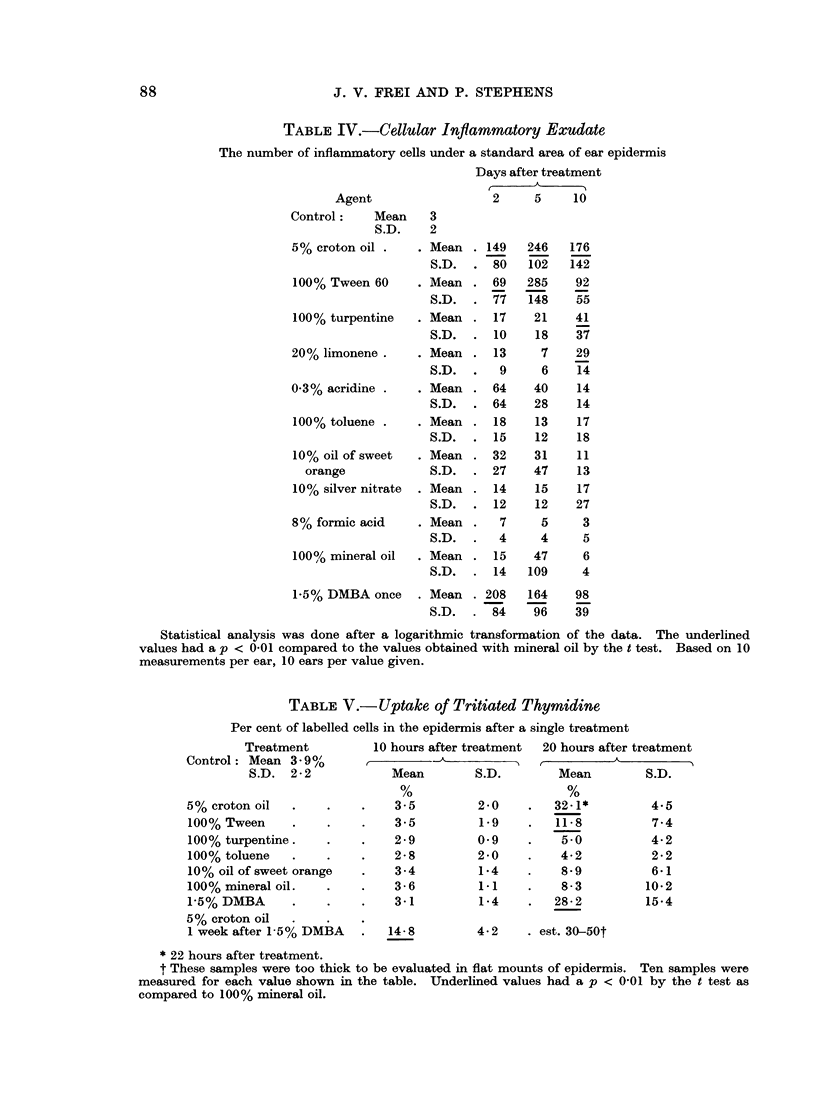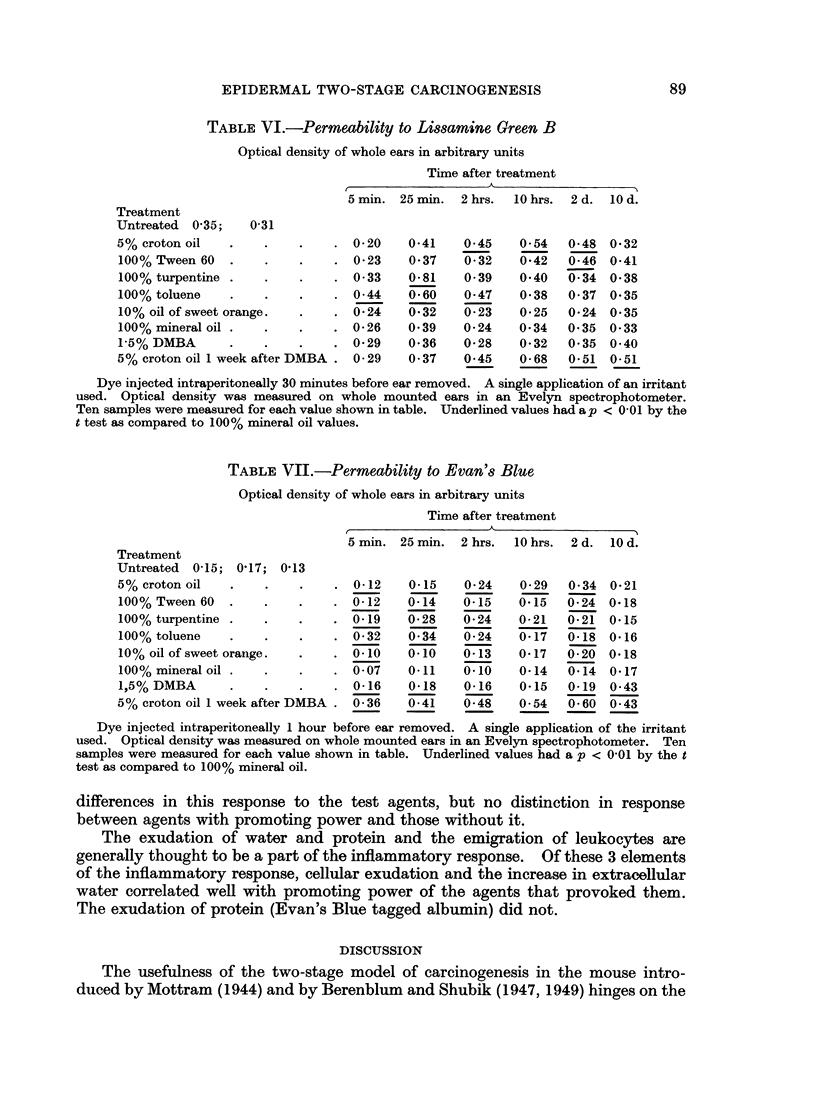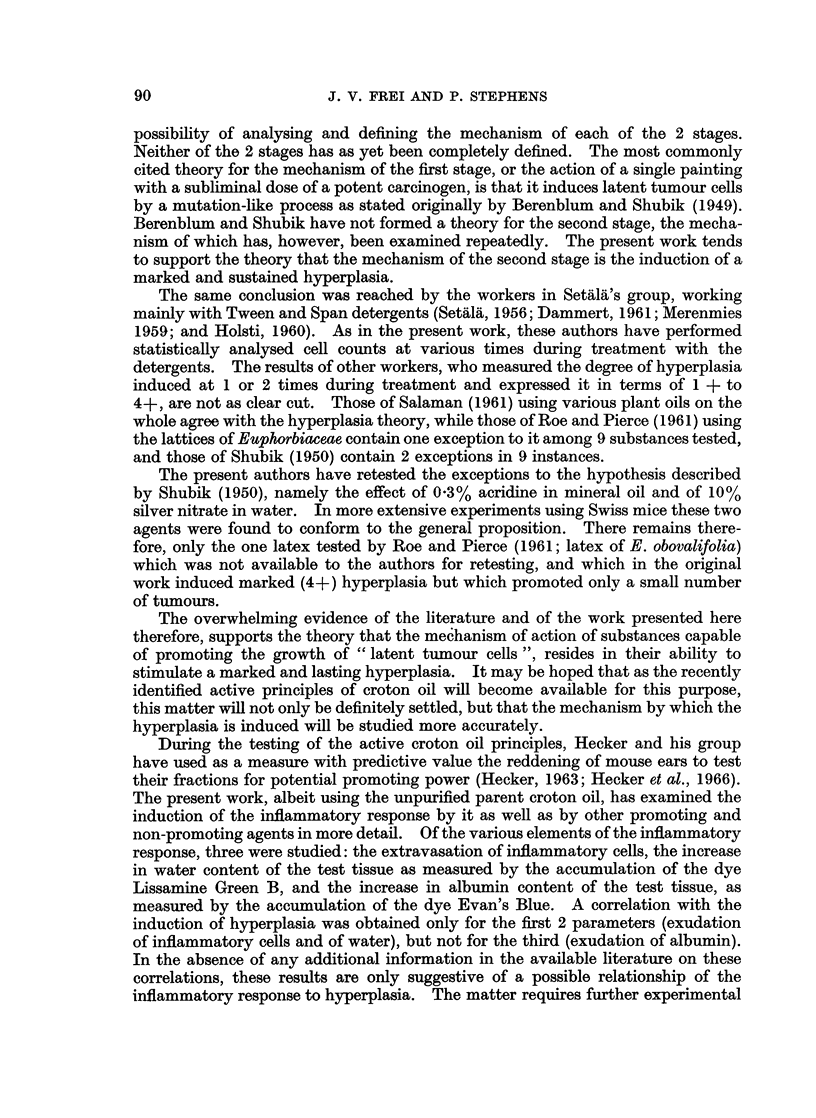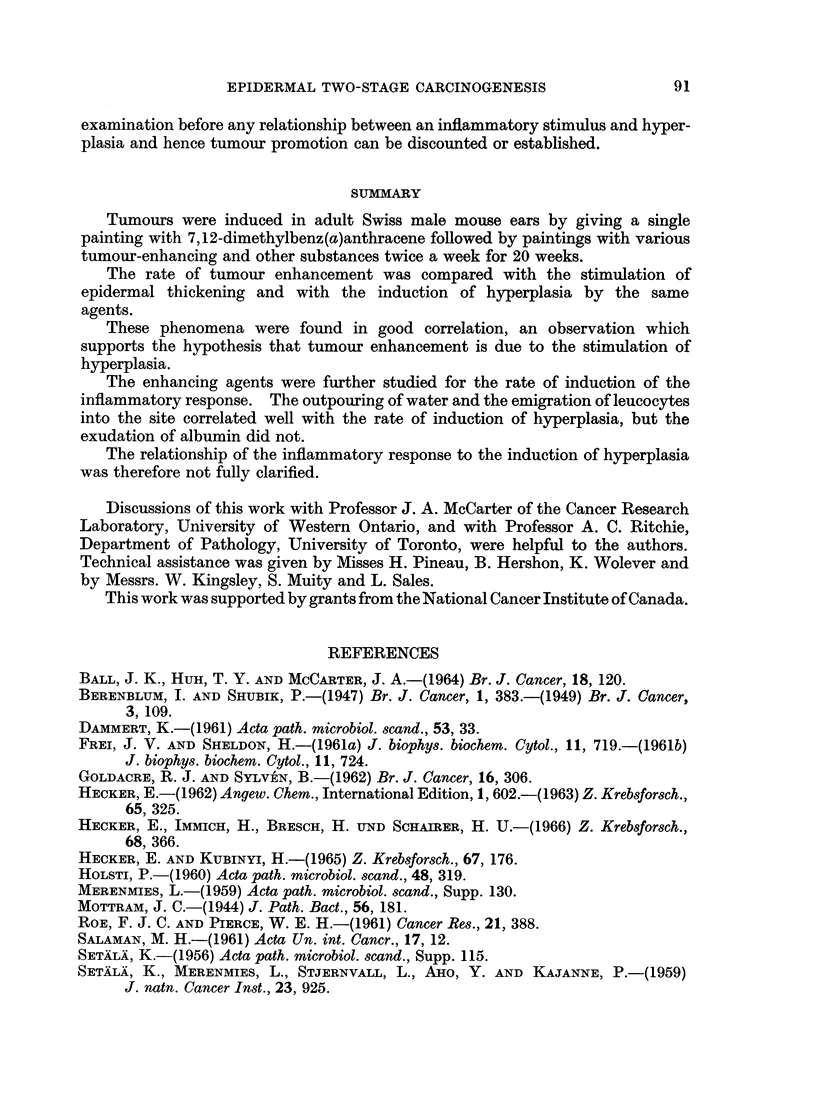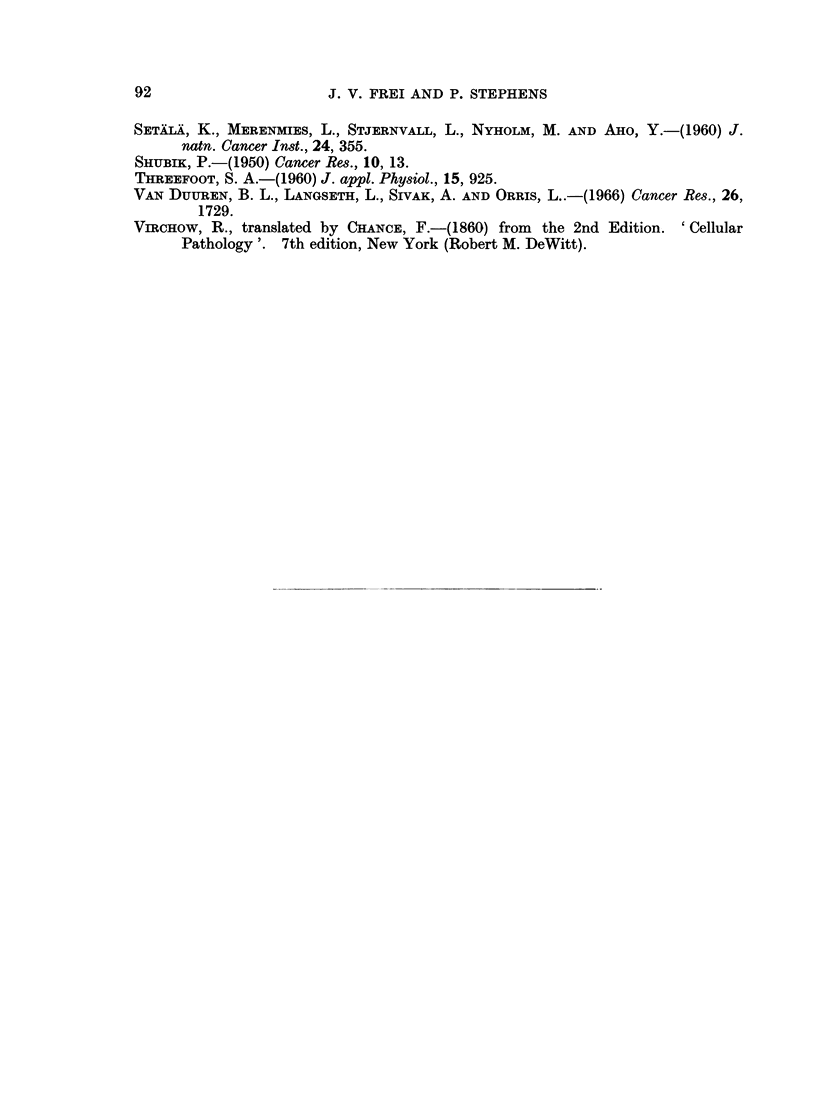# The correlation of promotion of tumour growth and of induction of hyperplasia in epidermal two-stage carcinogenesis.

**DOI:** 10.1038/bjc.1968.12

**Published:** 1968-03

**Authors:** J. V. Frei, P. Stephens


### THE CORRELATION OF PROMOTION OF TUMOUR GROWTH AND OF INDUCTION OF HYPERPLASIA IN EPIDERMAL TWO-STAGE CARCINOGENESIS

J. V. FREI* AND P. STEPHENS†

*From the Department of Pathology, Faculty of Medicine, McGill University, Montreal, Canada*

Received for publication October 30, 1967

MOTTRAM (1944) and Berenblum and Shubik (1949) have demonstrated the cocarcinogenic potency of croton oil applied topically to mouse skin which had been pretreated once with a dose of a carcinogenic polycyclic hydrocarbon too low to induce tumours. The active principles of croton oil responsible for this effect were more recently identified by the groups of Hecker (1962), Hecker and Kubinyi (1965) and of Van Duuren *et al.* (1966). The mechanism of action of these active principles or of their parent mixture is not yet known, though the work of Setälä *et. al.* (1959) and Merenmies (1959) on similarly cocarcinogenic Tween 60 and allied agents suggests that stimulation of cell proliferation may be an important feature of their action.

In this paper we examine the cocarcinogenic and cell proliferation stimulating actions of croton oil, of Tween 60 and of other agents, as well as the changes induced by these agents in the tissue, changes which may be part of their cocarcinogenic action.

### MATERIALS AND METHODS

The mice used in the experiments were 6–10 week old Swiss mice randomly bred in our laboratory. In one experiment inbred Swiss mice (Ball, Huh and McCarter, 1964) originally obtained from J. A. McCarter were used. The mice were kept 10 to a cage. The chemicals obtained commercially were used without additional purification. They were 7, 12-dimethylbenz (*a*) anthracene (DMBA) (Eastman Organic Chemicals, Rochester, N.Y.), croton oil (Boots Pure Drugs, Nottingham, U.K.). Tween 60 (Atlas Powder Co. Canada Ltd., Brantford, Ontario), toluene (Shell Oil Canada Ltd., Montreal), turpentine (Record Chemical Co., Inc,. Montreal), oil of sweet orange (R. D. Webb & Co., Inc., Linden, New Jersey), d-limonene (Eastman Organic Chemicals, Rochester, N.Y.), silver nitrate (Johnson, Matthey, & Mallory Ltd., Montreal), acridine (Eastman Organic Chemicals, Rochester, N.Y.), formic acid (Fisher Scientific Co., Montreal) and mineral oil (Nujol, Plough, Canada Ltd., Toronto). Some agents were used undiluted, most were dissolved in mineral oil, and formic acid and silver nitrate were dissolved in distilled water.

All the experiments were performed on male mouse ears. The test solutions were applied to the ears by means of a paint brush dipped into the solution. Both

* Present address: Cancer Research Laboratory, University of Western Ontario, London, Ontario, Canada.

† Present address: Department of Pathology, Medical College of Virginia, Richmond, Virginia. U.S.A.

sides of both ears were painted. No attempt was made to control the spreading of the solution to the adjacent skin, nor to determine the exact amount of material delivered to the ears. Nevertheless, brushes of the same design and constant conditions of painting were used throughout all the experiments.

Tumours were induced by painting the ears of groups of 30 or 60 mice once with 1·5% DMBA (initiation) followed beginning 1 week later by twice weekly paintings with the test solution (promotion) for 20 weeks. Any lesion larger than 1 mm. in diameter which was seen for at least 3 consecutive weeks, was considered to be a tumour. Tumours appearing towards the end of the experiment were observed for an additional 1 or 2 weeks to satisfy this criterion. Lesions which were first seen but later failed to be noted (" regressing " tumours) were not considered to be tumours.

For histological studies groups of 10 ears were removed in a standard manner close to the head of the anaesthetised animal, fixed in 10% formalin, and embedded in paraffin. Sections were cut as perpendicularly to the surface of the ears as possible and stained with haematoxylin and eosin. Measurements of epidermal and dermal changes were made on the outer surface of the ears, no closer than one high-power field from the edge of the ears. On each ear 10 areas of constant standard length of interfollicular epidermis were examined. By the use of an ocular counting grid, the number of nuclei in each area, the width of the area including the strata basale, spinosum and granuosulm, and the number of inflammatory cells under the area down to the auricular cartilage were determined separately. The values thus obtained were compared to those obtained for animals treated with mineral oil alone using the *t* test.

Autoradiography was performed on ears of mice given 2 $\mu$Ci of tritiated thymidine (Schwarz BioResearch) per g. body weight intraperitoneally 1 hour before sampling. The time of sampling was 10 a.m. Sheets of epidermis were removed by sharp dissection from the ears soaked for 5 hours in 0·5% acetic acid at 4° C. The sheets were glued to slides basal cell layer up and dipped in Ilford L4 emulsion. After a month's exposure the slides were developed, stained with haematoxylin and coverslipped. The proportion of labelled nuclei among 1000 nuclei of interfollicular epidermis was determined in each specimen. Only nuclei covered by more than 4 grains were considered to be labelled.

The uptake of the dyes Lissamine Green B or Evan's Blue was measured in treated ears by mounting whole ears in an Evelyn colourimeter. Lissamine Green B was injected intraperitoneally 30 minutes before sampling, whereas Evan's Blue was injected by the same route 1 hour before sampling. The ears were removed by the standard method described above and mounted carefully over an ear-shaped opening in a specially made sheet metal screen which fitted into the colorimeter. The opening was somewhat smaller than the ear. Optical density was then read against appropriate blanks. Ten ears were examined in each treatment group and compares for significance with respect to ears treated with mineral oil by the *t* test.

### RESULTS

Table I shows the result of a tumour-promotion experiment. Five per cent croton oil, full strength Tween 60 and full strength turpentine promoted significantly more tumours than appeared in the control group promoted with mineral oil. The yield of tumours with croton oil and with Tween 60 was almost identical but that with turpentine was considerably less. In these experiments full strength toluene and 10% of sweet orange promoted a yield of tumours no different from the controls.

A larger list of agents was tested for their ability to induce histologically measurable changes. Groups of 10 inbred Swiss mice were treated twice a week with the various agents, but without a preliminary DMBA treatment, except where specified. A set of 10 ears was removed, 2, 5, 10, 20 or 50 days from the beginning of the treatments and perpendicular sections were examined for increased epidermal cell number (Table II), for variations in thickness of the epidermis (Table III), and for the presence of a cellular inflammatory exudate (Table IV). It should be noted that in each case statistical analysis using the *t* test was done by comparing the treated ears to the appropriate groups treated with the solvent mineral oil alone.

TABLE I.—*Tumour Promotion*

| | | Results at 20 weeks of promotion | | | |
|---|---|---|---|---|---|
| Initiation with 1·5% DMBA | Promotion twice a week | No. of survivors | Tumour-bearing survivors | Tumours present | Tumours per survivor |
| Yes* | No | 23 | 4% | 1‖ | 0·04 |
| Yes† | 5% croton oil | 33 | 88% | 352‡ | 10·7 |
| Yes† | 100% Tween 60 | 41 | 95% | 345‡ | 8·4 |
| Yes* | 100% turpentine | 21 | 71% | 46‡ | 2·2 |
| Yes† | 100% toluene | 35 | 11% | 7‖ | 0·2 |
| Yes† | 10% oil of sweet orange | 52 | 8% | 7‖ | 0·13 |
| Yes† | 100% mineral oil | 53 | 11% | 8 | 0·15 |
| No* | 5% croton oil | 20 | 5% | 1‖ | 0·05 |
| No* | 100% Tween 60 | 25 | 0% | 0‖ | 0·0 |
| No* | 100% turpentine | 18 | 7% | 1‖ | 0·06 |
| No* | 100% toluene | 14 | 0% | 0‖ | 0·0 |
| No* | 10% oil of sweet orange | 24 | 0% | 0‖ | 0·0 |

* 30 mice at the start.
† 60 mice at the start.
‡ Yield significantly different at the 1% level from group promoted with mineral oil.
‖ Yield not significantly different.

Hyperplasia was defined by Virchow (1860) as growth of tissue by " numerical hypertrophy ", i.e. by an increase in the number of the constituent cells. The marked hyperplasia following repeated applications of croton oil or Tween 60 (Table II) is much like that seen by Merenmies (1959) with Tween 60. The number of cells rises rapidly and by the 10th day becomes stable at a new level. There is little difference between the effect of croton oil and Tween 60, or between croton oil with DMBA pretreatment and croton oil without it. Significant but less pronounced and less lasting hyperplasia was induced also by turpentine and by 20% limonene. We did not test limonene for promoting power and there is no record of its use as such in the literature.

The accompanying changes in the thickness of the epidermis seen in Table III are similar to those in hyperplasia. The thickness increases strikingly and markedly during the treatment with croton oil with or without DMBA pretreatment and during the treatment with Tween 60. The increase in thickness is about twice as large as the increase in the number of cells, so that true increase in cell size occurred as well. This is " true hypertrophy " as defined by Virchow (1860).

TABLE II.—*Hyperplasia*

Number of nuclei per standard legnth of a perpendicular cross-section of epidermis

| Agent | | | Days after treatment 2 | 5 | 10 | 20 | 50 |
|---|---|---|---|---|---|---|---|
| Control . | Mean | 136 | | | | | |
| | S.D. | 20·2 | | | | | |
| 5% croton oil . | Mean | | 197 | 255 | 286 | 283 | 286 |
| | S.D. | | 25·7 | 46·9 | 46·9 | 37·5 | 63·1 |
| 100% Tween 60 | Mean | | 154 | 228 | 213 | 204 | 217 |
| | S.D. | | 25·7 | 22·2 | 19·0 | 31·0 | 28·8 |
| 100% turpentine | Mean | | 141 | 141 | 174 | 169 | 212 |
| | S.D. | | 20·2 | 17·0 | 21·4 | 18·2 | 37·7 |
| 20% limonene . | Mean | | 127 | 131 | 172 | 146 | 198 |
| | S.D. | | 16·8 | 10·5 | 15·2 | 12·8 | 51·9 |
| 0·3% acridine . | Mean | | 146 | 168 | 151 | 180 | 228 |
| | S.D. | | 17·5 | 29·1 | 19·6 | 28·1 | 54·7 |
| 100% toluene . | Mean | | 149 | 138 | 137 | 158 | 167 |
| | S.D. | | 21·1 | 19·4 | 18·4 | 26·9 | 16·8 |
| 10% oil of sweet orange | Mean | | 161 | 160 | 148 | 159 | 187 |
| | S.D. | | 22·5 | 27·3 | 20·3 | 22·9 | 40·7 |
| 10% silver nitrate | Mean | | 126 | 142 | 148 | 128 | 142 |
| | S.D. | | 19·3 | 20·6 | 41·2 | 14·1 | 23·9 |
| 8% formic acid | Mean | | 127 | 130 | 120 | 159 | 144 |
| | S.D. | | 11·9 | 14·4 | 22·4 | 33·9 | 14·5 |
| 100% mineral oil | Mean | | 135 | 152 | 132 | 151 | 171 |
| | S.D. | | 13·6 | 36·8 | 14·9 | 14·5 | 27·4 |
| 1·5% DMBA once +5% croton oil | Mean | | 205 | 273 | 262 | 305 | 354 |
| | S.D. | | 48·7 | 70·3 | 39·9 | 54·4 | 77·5 |

Underlined values have a $p < 0{\cdot}01$ compared by the *t* test with the values obtained with mineral oil. Based on 10 measurements per ear, 10 ears per value given.

Electron micrographs of Setälä *et al.* (1960) using Tween 60 show that less than about one fifth of the area during hyperplasia is due to the opening up of the extracellular space. Similar observations can be made on our electron micrographs of treated epidermis (Frei and Sheldon, 1961*a*, 1961*b*).

Of the other agents only turpentine and acridine gave significant increase in the thickness of the epidermis at 2 of the times examined each and oil of sweet orange and limonene at 1 time each. We had not tested acridine for promoting power. Of the agents so tested, the weekly promoting turpentine gives the highest score both in terms of hyperplasia and in terms of increased thickness of the epidermis.

The results obtained in measuring the cellular inflammatory exudate (Table IV) are similar. The exudated cells were mostly neutrophils in all instances, but differential counts were not attempted. Croton oil with or without DMBA pre-treatment and Tween 60 gave a significantly greater formation of cellular exudate at all 3 times measured than the mineral oil controls. Turpentine and limonene gave a single significantly high value each and of the 2 agents turpentine was tested for promoting power as noted and proved to have some.

The next set of experiments was done using a shorter list of agents, which were applied *once only*. The uptake of tritiated thymidine (Table V) was increased

TABLE III.—*Thickness of Epidermis*

Arbitrary units of an ocular counting grid

| | | | Days after treatment | | | | |
|---|---|---|---|---|---|---|---|
| | | | 2 | 5 | 10 | 20 | 50 |
| Control . | Mean | 25·1 | | | | | |
| | S.D. | 7·4 | | | | | |
| 5% croton oil . | . Mean . | | 60 | 114 | 123 | 122 | 106 |
| | S.D. . | | 10·2 | 28·8 | 20·2 | 27·6 | 35·3 |
| 100% Tween 60 | . Mean . | | 43 | 91 | 82 | 67 | 64 |
| | S.D. . | | 10·2 | 14·2 | 10·7 | 9·1 | 9·6 |
| 100% turpentine | . Mean . | | 26 | 33 | 37 | 42 | 71 |
| | S.D. . | | 4·9 | 11·4 | 10·7 | 13·7 | 20·5 |
| 20% limonene . | . Mean . | | 25 | 27 | 39 | 30 | 46 |
| | S.D. . | | 4·8 | 7·3 | 6·7 | 5·7 | 18·9 |
| 0·3% acridine . | . Mean . | | 38 | 41 | 35 | 44 | 73 |
| | S.D. . | | 8·3 | 10·1 | 8·9 | 11·0 | 26·2 |
| 100% toluene . | . Mean . | | 27 | 26 | 26 | 31 | 38 |
| | S.D. . | | 6·9 | 6·6 | 7·1 | 7·6 | 6·5 |
| 10% oil of sweet orange | . Mean . | | 37 | 35 | 36 | 34 | 45 |
| | S.D. . | | 6·2 | 10·1 | 5·6 | 8·5 | 13·4 |
| 10% silver nitrate | . Mean . | | 24 | 28 | 32 | 24 | 25 |
| | S.D. . | | 6·2 | 9·0 | 17·0 | 3·0 | 5·4 |
| 8% formic acid | . Mean . | | 20 | 22 | 24 | 28 | 26 |
| | S.D. . | | 3·8 | 5·2 | 6·2 | 9·6 | 5·4 |
| 100% mineral oil | . Mean . | | 30 | 36 | 25 | 33 | 43 |
| | S.D. . | | 6·9 | 22·0 | 5·2 | 6·5 | 16·8 |
| 1·5% DMBA once | . Mean . | | 76 | 127 | 103 | 107 | 139 |
| +5% croton oil | . S.D. . | | 19·5 | 27·8 | 21·5 | 33·7 | 29·9 |

Underlined values have a $p < 0{\cdot}01$ compared by the $t$ test with the values obtained with mineral oil. Based on 10 measurements per ear, 10 ears per value given.

by croton oil and by Tween 60 after a lag period of about 10 hours. DMBA had the same effect. The other agents tested did not increase thymidine uptake for at least 20 hours following treatment to a significant degree compared with mineral oil. This observation does not exclude the possibility that significantly increased uptakes may be observed by these other agents at other times following an application. Nevertheless, the 2 powerful promoting agents croton oil and Tween 60 were found to stimulate DNA synthesis markedly 20 hours following an application, as their effectiveness in inducing hyperplasia suggested they should.

The dye Lissamine Green B may be used to measure the distribution of extracellular water (Goldacre and Sylvén, 1962). Table VI demonstrates that extracellular water is markedly but transiently increased soon after the application of turpentine and toluene, but that it is increased late and for a longer period of time following the application of croton oil with or without DMBA pretreatment or of Tween 60. The early transient rise does not correlate with the promoting activity of the 2 agents that caused it. The delayed rise, on the other hand, is present only with the powerful promoters croton oil and Tween 60.

The dye Evan's Blue is thought to become attached to albumin and to measure approximately albumin distribution (Threefoot, 1960; Table VII). We found

TABLE IV.—*Cellular Inflammatory Exudate*

The number of inflammatory cells under a standard area of ear epidermis

| Agent | | | Days after treatment 2 | 5 | 10 |
|---|---|---|---|---|---|
| Control : | Mean | 3 | | | |
| | S.D. | 2 | | | |
| 5% croton oil | Mean | | 149 | 246 | 176 |
| | S.D. | | 80 | 102 | 142 |
| 100% Tween 60 | Mean | | 69 | 285 | 92 |
| | S.D. | | 77 | 148 | 55 |
| 100% turpentine | Mean | | 17 | 21 | 41 |
| | S.D. | | 10 | 18 | 37 |
| 20% limonene | Mean | | 13 | 7 | 29 |
| | S.D. | | 9 | 6 | 14 |
| 0·3% acridine | Mean | | 64 | 40 | 14 |
| | S.D. | | 64 | 28 | 14 |
| 100% toluene | Mean | | 18 | 13 | 17 |
| | S.D. | | 15 | 12 | 18 |
| 10% oil of sweet orange | Mean | | 32 | 31 | 11 |
| | S.D. | | 27 | 47 | 13 |
| 10% silver nitrate | Mean | | 14 | 15 | 17 |
| | S.D. | | 12 | 12 | 27 |
| 8% formic acid | Mean | | 7 | 5 | 3 |
| | S.D. | | 4 | 4 | 5 |
| 100% mineral oil | Mean | | 15 | 47 | 6 |
| | S.D. | | 14 | 109 | 4 |
| 1·5% DMBA once | Mean | | 208 | 164 | 98 |
| | S.D. | | 84 | 96 | 39 |

Statistical analysis was done after a logarithmic transformation of the data. The underlined values had a $p < 0{\cdot}01$ compared to the values obtained with mineral oil by the $t$ test. Based on 10 measurements per ear, 10 ears per value given.

TABLE V.—*Uptake of Tritiated Thymidine*

Per cent of labelled cells in the epidermis after a single treatment

| Treatment | 10 hours after treatment Mean % | S.D. | 20 hours after treatment Mean % | S.D. |
|---|---|---|---|---|
| Control : Mean 3·9% S.D. 2·2 | | | | |
| 5% croton oil | 3·5 | 2·0 | 32·1* | 4·5 |
| 100% Tween | 3·5 | 1·9 | 11·8 | 7·4 |
| 100% turpentine | 2·9 | 0·9 | 5·0 | 4·2 |
| 100% toluene | 2·8 | 2·0 | 4·2 | 2·2 |
| 10% oil of sweet orange | 3·4 | 1·4 | 8·9 | 6·1 |
| 100% mineral oil | 3·6 | 1·1 | 8·3 | 10·2 |
| 1·5% DMBA | 3·1 | 1·4 | 28·2 | 15·4 |
| 5% croton oil 1 week after 1·5% DMBA | 14·8 | 4·2 | est. 30–50† | |

* 22 hours after treatment.

† These samples were too thick to be evaluated in flat mounts of epidermis. Ten samples were measured for each value shown in the table. Underlined values had a $p < 0{\cdot}01$ by the $t$ test as compared to 100% mineral oil.

TABLE VI.—*Permeability to Lissamine Green B*

Optical density of whole ears in arbitrary units

| Treatment | Time after treatment | | | | | |
|---|---|---|---|---|---|---|
| | 5 min. | 25 min. | 2 hrs. | 10 hrs. | 2 d. | 10 d. |
| Untreated 0·35; 0·31 | | | | | | |
| 5% croton oil | 0·20 | 0·41 | 0·45 | 0·54 | 0·48 | 0·32 |
| 100% Tween 60 | 0·23 | 0·37 | 0·32 | 0·42 | 0·46 | 0·41 |
| 100% turpentine | 0·33 | 0·81 | 0·39 | 0·40 | 0·34 | 0·38 |
| 100% toluene | 0·44 | 0·60 | 0·47 | 0·38 | 0·37 | 0·35 |
| 10% oil of sweet orange | 0·24 | 0·32 | 0·23 | 0·25 | 0·24 | 0·35 |
| 100% mineral oil | 0·26 | 0·39 | 0·24 | 0·34 | 0·35 | 0·33 |
| 1·5% DMBA | 0·29 | 0·36 | 0·28 | 0·32 | 0·35 | 0·40 |
| 5% croton oil 1 week after DMBA | 0·29 | 0·37 | 0·45 | 0·68 | 0·51 | 0·51 |

Dye injected intraperitoneally 30 minutes before ear removed. A single application of an irritant used. Optical density was measured on whole mounted ears in an Evelyn spectrophotometer. Ten samples were measured for each value shown in table. Underlined values had a $p < 0 \cdot 01$ by the $t$ test as compared to 100% mineral oil values.

TABLE VII.—*Permeability to Evan's Blue*

Optical density of whole ears in arbitrary units

| Treatment | Time after treatment | | | | | |
|---|---|---|---|---|---|---|
| | 5 min. | 25 min. | 2 hrs. | 10 hrs. | 2 d. | 10 d. |
| Untreated 0·15; 0·17; 0·13 | | | | | | |
| 5% croton oil | 0·12 | 0·15 | 0·24 | 0·29 | 0·34 | 0·21 |
| 100% Tween 60 | 0·12 | 0·14 | 0·15 | 0·15 | 0·24 | 0·18 |
| 100% turpentine | 0·19 | 0·28 | 0·24 | 0·21 | 0·21 | 0·15 |
| 100% toluene | 0·32 | 0·34 | 0·24 | 0·17 | 0·18 | 0·16 |
| 10% oil of sweet orange | 0·10 | 0·10 | 0·13 | 0·17 | 0·20 | 0·18 |
| 100% mineral oil | 0·07 | 0·11 | 0·10 | 0·14 | 0·14 | 0·17 |
| 1,5% DMBA | 0·16 | 0·18 | 0·16 | 0·15 | 0·19 | 0·43 |
| 5% croton oil 1 week after DMBA | 0·36 | 0·41 | 0·48 | 0·54 | 0·60 | 0·43 |

Dye injected intraperitoneally 1 hour before ear removed. A single application of the irritant used. Optical density was measured on whole mounted ears in an Evelyn spectrophotometer. Ten samples were measured for each value shown in table. Underlined values had a $p < 0 \cdot 01$ by the $t$ test as compared to 100% mineral oil.

differences in this response to the test agents, but no distinction in response between agents with promoting power and those without it.

The exudation of water and protein and the emigration of leukocytes are generally thought to be a part of the inflammatory response. Of these 3 elements of the inflammatory response, cellular exudation and the increase in extracellular water correlated well with promoting power of the agents that provoked them. The exudation of protein (Evan's Blue tagged albumin) did not.

### DISCUSSION

The usefulness of the two-stage model of carcinogenesis in the mouse introduced by Mottram (1944) and by Berenblum and Shubik (1947, 1949) hinges on the possibility of analysing and defining the mechanism of each of the 2 stages. Neither of the 2 stages has as yet been completely defined. The most commonly cited theory for the mechanism of the first stage, or the action of a single painting with a subliminal dose of a potent carcinogen, is that it induces latent tumour cells by a mutation-like process as stated originally by Berenblum and Shubik (1949). Berenblum and Shubik have not formed a theory for the second stage, the mechanism of which has, however, been examined repeatedly. The present work tends to support the theory that the mechanism of the second stage is the induction of a marked and sustained hyperplasia.

The same conclusion was reached by the workers in Setälä's group, working mainly with Tween and Span detergents (Setälä, 1956; Dammert, 1961; Merenmies 1959; and Holsti, 1960). As in the present work, these authors have performed statistically analysed cell counts at various times during treatment with the detergents. The results of other workers, who measured the degree of hyperplasia induced at 1 or 2 times during treatment and expressed it in terms of 1 + to 4+, are not as clear cut. Those of Salaman (1961) using various plant oils on the whole agree with the hyperplasia theory, while those of Roe and Pierce (1961) using the lattices of *Euphorbiaceae* contain one exception to it among 9 substances tested, and those of Shubik (1950) contain 2 exceptions in 9 instances.

The present authors have retested the exceptions to the hypothesis described by Shubik (1950), namely the effect of 0·3% acridine in mineral oil and of 10% silver nitrate in water. In more extensive experiments using Swiss mice these two agents were found to conform to the general proposition. There remains therefore, only the one latex tested by Roe and Pierce (1961; latex of *E. obovalifolia*) which was not available to the authors for retesting, and which in the original work induced marked (4+) hyperplasia but which promoted only a small number of tumours.

The overwhelming evidence of the literature and of the work presented here therefore, supports the theory that the mechanism of action of substances capable of promoting the growth of "latent tumour cells", resides in their ability to stimulate a marked and lasting hyperplasia. It may be hoped that as the recently identified active principles of croton oil will become available for this purpose, this matter will not only be definitely settled, but that the mechanism by which the hyperplasia is induced will be studied more accurately.

During the testing of the active croton oil principles, Hecker and his group have used as a measure with predictive value the reddening of mouse ears to test their fractions for potential promoting power (Hecker, 1963; Hecker *et al.*, 1966). The present work, albeit using the unpurified parent croton oil, has examined the induction of the inflammatory response by it as well as by other promoting and non-promoting agents in more detail. Of the various elements of the inflammatory response, three were studied: the extravasation of inflammatory cells, the increase in water content of the test tissue as measured by the accumulation of the dye Lissamine Green B, and the increase in albumin content of the test tissue, as measured by the accumulation of the dye Evan's Blue. A correlation with the induction of hyperplasia was obtained only for the first 2 parameters (exudation of inflammatory cells and of water), but not for the third (exudation of albumin). In the absence of any additional information in the available literature on these correlations, these results are only suggestive of a possible relationship of the inflammatory response to hyperplasia. The matter requires further experimental examination before any relationship between an inflammatory stimulus and hyperplasia and hence tumour promotion can be discounted or established.

### SUMMARY

Tumours were induced in adult Swiss male mouse ears by giving a single painting with 7,12-dimethylbenz(*a*)anthracene followed by paintings with various tumour-enhancing and other substances twice a week for 20 weeks.

The rate of tumour enhancement was compared with the stimulation of epidermal thickening and with the induction of hyperplasia by the same agents.

These phenomena were found in good correlation, an observation which supports the hypothesis that tumour enhancement is due to the stimulation of hyperplasia.

The enhancing agents were further studied for the rate of induction of the inflammatory response. The outpouring of water and the emigration of leucocytes into the site correlated well with the rate of induction of hyperplasia, but the exudation of albumin did not.

The relationship of the inflammatory response to the induction of hyperplasia was therefore not fully clarified.

Discussions of this work with Professor J. A. McCarter of the Cancer Research Laboratory, University of Western Ontario, and with Professor A. C. Ritchie, Department of Pathology, University of Toronto, were helpful to the authors. Technical assistance was given by Misses H. Pineau, B. Hershon, K. Wolever and by Messrs. W. Kingsley, S. Muity and L. Sales.

This work was supported by grants from the National Cancer Institute of Canada.

### REFERENCES

Ball, J. K., Huh, T. Y. and McCarter, J. A.—(1964) *Br. J. Cancer*, **18**, 120.

Berenblum, I. and Shubik, P.—(1947) *Br. J. Cancer*, **1**, 383.—(1949) *Br. J. Cancer*, **3**, 109.

Dammert, K.—(1961) *Acta path. microbiol. scand.*, **53**, 33.

Frei, J. V. and Sheldon, H.—(1961*a*) *J. biophys. biochem. Cytol.*, **11**, 719.—(1961*b*) *J. biophys. biochem. Cytol.*, **11**, 724.

Goldacre, R. J. and Sylvén, B.—(1962) *Br. J. Cancer*, **16**, 306.

Hecker, E.—(1962) *Angew. Chem.*, International Edition, **1**, 602.—(1963) *Z. Krebsforsch.*, **65**, 325.

Hecker, E., Immich, H., Bresch, H. und Schairer, H. U.—(1966) *Z. Krebsforsch.*, **68**, 366.

Hecker, E. and Kubinyi, H.—(1965) *Z. Krebsforsch.*, **67**, 176.

Holsti, P.—(1960) *Acta path. microbiol. scand.*, **48**, 319.

Merenmies, L.—(1959) *Acta path. microbiol. scand.*, Supp. 130.

Mottram, J. C.—(1944) *J. Path. Bact.*, **56**, 181.

Roe, F. J. C. and Pierce, W. E. H.—(1961) *Cancer Res.*, **21**, 388.

Salaman, M. H.—(1961) *Acta Un. int. Cancr.*, **17**, 12.

Setälä, K.—(1956) *Acta path. microbiol. scand.*, Supp. 115.

Setälä, K., Merenmies, L., Stjernvall, L., Aho, Y. and Kajanne, P.—(1959) *J. natn. Cancer Inst.*, **23**, 925.

SETÄLÄ, K., MERENMIES, L., STJERNVALL, L., NYHOLM, M. AND AHO, Y.—(1960) *J. natn. Cancer Inst.*, **24**, 355.
SHUBIK, P.—(1950) *Cancer Res.*, **10**, 13.
THREEFOOT, S. A.—(1960) *J. appl. Physiol.*, **15**, 925.
VAN DUUREN, B. L., LANGSETH, L., SIVAK, A. AND ORRIS, L..—(1966) *Cancer Res.*, **26**, 1729.
VIRCHOW, R., translated by CHANCE, F.—(1860) from the 2nd Edition. 'Cellular Pathology'. 7th edition, New York (Robert M. DeWitt).